# National Medical Convention

**Published:** 1846-06

**Authors:** 


*National Medical Convention.*—The National Medical Convention at New York, having transacted all the business for which it was convened, adjourned to meet at Philadelphia on the first Wednesday in May next. The following resolution offered by Dr. O. S. Bartles, of New York, led to a protracted discussion in the convention, and was finally referred to a committee, with directions to report on the subject at the meeting in Philadelphia:

*Resolved*, That the union of the business of teaching and licensing, in the same hands, is wrong in principle, and liable to great abuse in practice. Instead of conferring the right to license on medical colleges, and State and county medical societies, it should be restricted to one board in each State, composed, in fair proportion, of representatives from the medical colleges, and the profession at large, and the pay for whose services, as examiners, should, in no degree, depend on the number licensed by them.

